# Patient perspectives and experiences of collaborative pharmacist prescribing in the hospital inpatient setting: a cross-sectional survey

**DOI:** 10.1007/s11096-026-02178-0

**Published:** 2026-06-27

**Authors:** Hana Amer, Sally Marotti, Jacinta Johnson, Imaina Widagdo, Kirsten Burns, Sharon Goldsworthy, Ramim Hossin, Molly Astley, Lisa Kalisch Ellett

**Affiliations:** 1 Quality Use of Medicines and Pharmacy Research Centre, School of Pharmacy and Biomedical Science, College of Health, Adelaide University, Adelaide, SA 5001 Australia; 2 SA Pharmacy, SA Health, L7 11 Hindmarsh Square, Adelaide, SA 5000 Australia; 3 Faculty of Medicine and Health, School of Pharmacy, The University of Sydney, Sydney, NSW 2050 Australia; 4 College of Medicine and Public Health, Flinders University, Adelaide, Australia

**Keywords:** Electronic prescribing, Hospital, Non-medical prescribing, Patient involvement, Patients, Pharmacists, Supplementary prescribing

## Abstract

**Introduction:**

Collaborative pharmacist prescribing in hospitals involves pharmacists, medical doctors, and patients working together to develop medicine plans that support shared decision-making and underpin pharmacist prescriptions. Shared decision-making is a process in which clinicians and patients make treatment decisions together, ensuring they reflect patient preferences and understanding. However, little is known about patient perspectives and experiences with collaborative pharmacist prescribing, particularly related to shared decision-making, in the inpatient hospital setting.

**Aim:**

To examine patient perspectives and experiences of collaborative pharmacist prescribing in the hospital inpatient setting.

**Method:**

A cross-sectional survey was conducted across four hospitals in South Australia. Eligible inpatients were aged ≥ 18 years and provided informed consent. The primary outcome was patient perspectives of collaborative pharmacist prescribing and experiences during medicine prescribing as an inpatient. Results were stratified by the type of prescribing model (collaborative pharmacist prescribing vs. medical prescribing) received by the patient during their admission. Free-text responses were thematically analysed using Braun and Clarke’s framework. Likert scale responses were summarised using descriptive statistics, and sub-group analysis compared categorical variables between groups.

**Results:**

Responses were received from 200 patients (100 who received collaborative pharmacist prescribing and 100 who received medical doctor prescribing). Overall, patients had a median age of 72 years and just over half were female. Qualitative analysis found that all respondents trust pharmacists’ expertise and ability to collaboratively prescribe. Patients in both groups perceived benefits of collaborative pharmacist prescribing including enhanced interprofessional communication, reductions in medication errors, and a better understanding of their medicines. These findings reinforced the quantitative results, with a higher proportion of patients who received collaborative pharmacist prescribing reporting feeling very or extremely involved in medicine decisions compared to patients who received medical prescribing (60% vs. 14%, *p* < 0.001).

**Conclusion:**

Collaborative pharmacist prescribing enhances shared decision-making by actively involving patients in discussions about their medicines. It improves patients’ understanding of their medicines, which supports safer and more informed treatment decisions. These findings support wider adoption of collaborative pharmacist prescribing to improve patient-centered care in hospitals.

**Supplementary Information:**

The online version contains supplementary material available at https://doi.org/10.1007/s11096-026-02178-0.

## Impact statements

- Embedding pharmacists in collaborative prescribing roles strengthens patient-centred care by actively involving patients in discussions about their medicines
- Collaborative pharmacist prescribing supports shared-decision making, helping patients feel more informed and confident about their medicines in hospital
- Integrating pharmacists into prescribing teams supports interprofessional collaboration and contributes to safer, more coordinated medication management in hospitals
- These findings support embedding pharmacists in collaborative prescribing roles to enhance patient-centred care, shared decision-making, and improve patient care

## Introduction

Collaborative pharmacist prescribing, also referred to as supplementary or partnered prescribing, has expanded across healthcare systems in countries such as Australia, The United States (US), Canada and United Kingdom (UK), as a strategy to improve medicine safety and optimise medicine use [1, 2]. These models involve pharmacists working with medical doctors and patients to develop medicine plans that underpin pharmacist prescribing. While the United Kingdom has largely transitioned towards independent pharmacist prescribing, collaborative models remain widely utilised in hospital inpatient settings, where multi-disciplinary teamwork is integral to patient care [3–6].

Evidence from collaborative pharmacist prescribing models have demonstrated a range of clinical benefits, including reductions in prescribing errors and medication-related harm [7–13]. Humanistic outcomes have also been reported, such as increased work satisfaction, enhanced interprofessional communication, and reductions in health professional workload [14, 15]. In addition, economic and system-level benefits have also been reported, including shorter hospital stays and reduced healthcare costs [16–18].

Understanding patients’ perspectives and experiences of collaborative pharmacist prescribing is essential to ensuring effective communication, fostering trust, and ensuring medicine plans are tailored to the patients' needs and preferences [19–21]. Despite the growing body of evidence supporting collaborative pharmacist prescribing, there remains limited evidence describing patient perspectives and experiences within inpatient hospital settings [22].

A 2018 systematic review identified generally positive patient perceptions of pharmacist prescribing, including high satisfaction and perceived safety, predominantly in primary and secondary care settings [19]. More recent qualitative studies indicate that patients trust pharmacists and value their medicine expertise, accessibility, and supportive roles, including medication education and psychosocial support in relation to collaborative prescribing models. These findings have been reported across both established and exploratory collaborative prescribing models, spanning a range of practice settings, including primary care, outpatient haemodialysis, and preoperative opioid-tapering services [19, 20, 23–26]. However, inpatient hospital care has largely been missing from prior studies investigating patient perceptions of pharmacist prescribing.

While prior studies have examined patient acceptability, trust, and satisfaction with pharmacist prescribing, the extent and nature of patients’ involvement in shared decision-making at the point of prescribing, is less understood. Partnering with consumers is a fundamental principle underpinning global health policy. Similarly, shared decision-making is widely viewed as the most important component of patient-centred care [27, 28]. It is a collaborative process in which healthcare professionals work with patients to make informed decisions about their healthcare [27, 28]. The World Health Organization advocates for people-centred health systems through its Framework on Integrated People-Centred Health Services, emphasising patient inclusion in care design and delivery [29]. National initiatives in the UK, USA and Australia promote shared decision-making as a mechanism to improve care quality, patient empowerment and safety [30–34].

Although collaborative pharmacist prescribing is increasingly being implemented in hospital settings, evidence describing patients’ perspectives and experiences, particularly their involvement in shared decision-making during inpatient care, remains limited. Understanding how patients perceive and experience collaborative pharmacist prescribing in acute hospital care is essential to inform ongoing service development and ensure that medicines management remains aligned within patient-centred care principles [35, 36].

In South Australia, a collaborative pharmacist prescribing model has been implemented within acute and general medical units within several public hospitals. The model has been previously described [7]. Briefly, prescribing pharmacists obtain and document a medication history on admission and work in partnership with the medical doctor and patient to develop an individualised medicine plan aligned with the patients' treatment goals. The plan is documented in the electronic medical record and endorsed by both the pharmacist and treating medical practitioner, after which pharmacist prescribing is undertaken in accordance with the plan. This model is embedded within routine multidisciplinary care in selected inpatient services, where collaborative decision-making and continuity of medication management are critical to patient safety. At the study sites pharmacists undertook collaborative pharmacist prescribing on admission and during inpatient care, for example during consultant ward rounds. However, at the time the study was conducted, discharge prescribing was restricted to medical doctors.

This study was guided by the Donabedian model of healthcare quality and the Patient-centred Medication Safety (P-MEDS) framework [37, 38]. Donabedian’s model conceptualises healthcare quality across structure, process, and outcomes, providing a widely used framework for examining quality of care [37]. The P-MEDS framework emphasises patient-centred medication safety through effective communication, interprofessional teamwork, and patient engagement, including shared decision-making [38].

### Aim

This study aimed to examine patient perspectives and experiences of collaborative pharmacist prescribing in the inpatient hospital setting.

## Method

### Study design

A cross-sectional anonymous survey was conducted and reported in accordance with the Strengthening the Reporting of Observational studies in Epidemiology (STROBE) checklist for cross-sectional studies and the Checklist for Reporting of Survey Studies (CROSS) [39, 40] (Supplementary Files 1, 2).

### Study setting

This study was conducted within four South Australian public hospitals. Three of the sites are metropolitan teaching hospitals, ranging in size from approximately 260 to 800 beds, while the fourth is a regional hospital with around 80 beds. All sites use the same electronic medical record, which incorporated electronic prescribing, (Sunrise™). Collaborative pharmacist prescribing is implemented within acute and general medical units at the hospital sites. Not all patients within these units receive pharmacist prescribing, with many receiving medical prescribing. Due to staffing constraints, pharmacists prioritise patients at high-risk of medication-related harm for the service (e.g. older adults, polypharmacy, high-risk medications), as they do for standard pharmacy services [7].

### Study participants

Patients admitted to acute or general medical units at participating hospitals between September 2023 and March 2025 were eligible to participate if they were aged ≥ 18 years and provided informed consent. Patients with limited English proficiency could participate if a translator, family member or friend could assist with questionnaire completion.

### Questionnaire development

Two versions of the questionnaire were developed, one for patients who received collaborative pharmacist prescribing and one for patients who received medical prescribing. Questionnaires were largely identical, with minor differences in wording and introductory text to reflect the prescribing model received (Supplementary File 3).

**Fig. 1:**
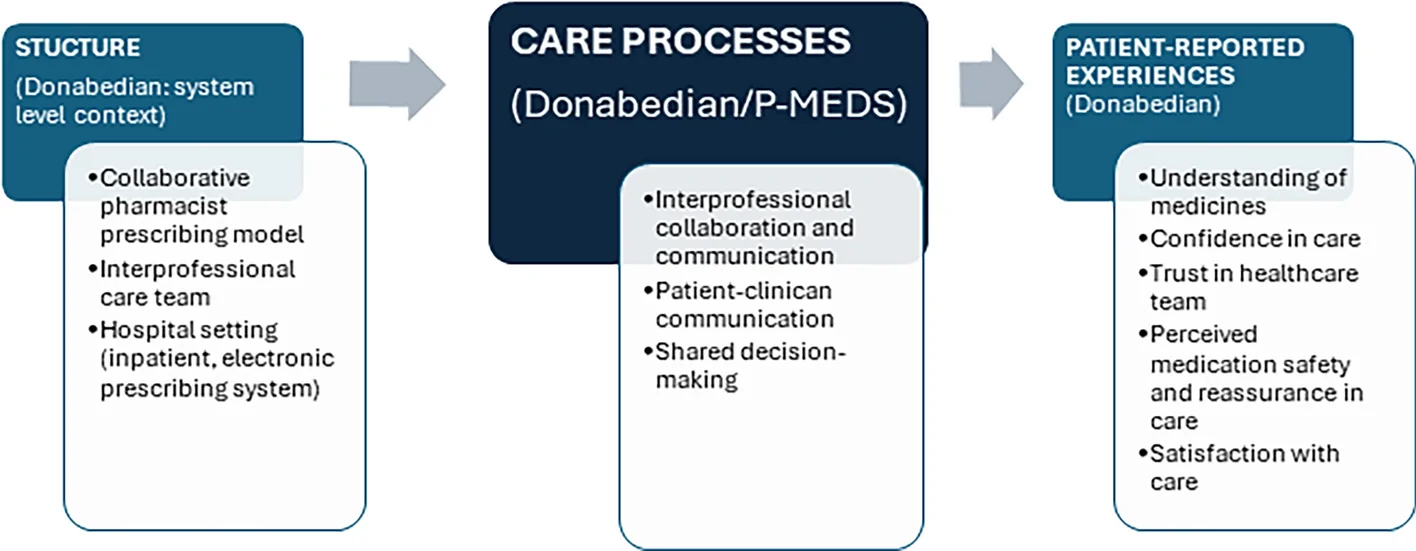
Conceptual framework underpinning the study

The study was informed by the Donabedian model of healthcare quality and the P-MEDS framework, which were used as conceptual frameworks to guide the development of survey items and interpretation of patient perceptions and experiences [37, 38] (Fig. 1). Donabedian’s model conceptualises healthcare quality across three interrelated domains: structure (organisational and professional arrangements), process (how care is delivered), and outcomes (the effects of care on patients) [37]. Consistent with the study aims, the survey focused primarily on process-of-care domains, including communication, interprofessional collaboration, and shared decision-making, as these represent key mechanisms through which collaborative pharmacist prescribing may influence patient experience and medication safety in hospital settings.

The P-MEDS framework further informed questionnaire development by framing collaborative pharmacist prescribing as a patient-centred and collaborative process shaped by effective communication, teamwork, and patient engagement [38]. Guided by this framework, survey items explored patient perceptions and experiences of pharmacist involvement in prescribing, understanding of their medicines, confidence in care, reassurance regarding medication safety, and trust in care provision. These domains align with the core components of the P-MEDS framework, including interprofessional teamwork, effective communication, patient engagement and shared decision-making. Questions assessing patient attitudes and perspectives towards collaborative pharmacist prescribing were adapted from prior research [21, 41].Demographic questions were also included.

Responses were collected using tick-boxes, five-point Likert scales (‘strongly agree’ to ‘strongly disagree’), and free-text. Two items assessed participants’ perceived and desired levels of involvement in shared decision-making using a five-point scale (‘extremely involved’ to ‘not at all involved’). Three free-text questions explored unanswered medication questions, perceived benefits of pharmacist prescribing, and views on pharmacist involvement in prescribing. The questionnaire underwent expert, consumer, and cultural review, followed by pilot testing with a convenience sample of consumers (n = 5), with no subsequent modifications. Final questionnaires are provided in Supplementary Files 3 and 4.

### Outcomes

The primary outcome was patient perspectives of collaborative pharmacist prescribing and experiences during medicine prescribing as an inpatient. Results were stratified by the type of prescribing model (collaborative pharmacist prescribing vs. medical prescribing) received by the patient during their admission.

### Survey administration

Patients were approached by a pharmacist, pharmacy technician, or pharmacy student to participate in the survey. Participants were provided with an information sheet which outlined the purposes of the study, as well the potential benefits, risks, privacy considerations, and their rights as participants. Consent was implied through completion and submission of the survey. Questionnaires were completed online via Qualtrics, on paper, or verbally, according to patient preference. To minimise bias, questionnaires were completed independently or with assistance from a family member or staff member not involved in prescribing their medicines. Verbal questionaries were administered by a healthcare professional independent of the patients’ prescribing who documented the free-text questions verbatim. For online completion, Qualtrics settings were used to restrict multiple submissions from the same device.

### Sampling strategy

As the number of patients receiving collaborative pharmacist prescribing was relatively limited, all patients who received collaborative pharmacist prescribing during the study period were eligible to participate if they were able to provide informed consent to maximise recruitment from this group. Patients who received medical prescribing were recruited from the same clinical units as patients who received collaborative pharmacist prescribing. Eligible patients were selected using a random number generator and were invited to participate [42]. We aimed to obtain approximately equal numbers of survey responses from each hospital, where feasible, recognising potential differences in patient flow and recruitment rates across sites. To achieve balanced recruitment across prescribing groups, the surveys were closed once 100 responses were obtained from each prescribing group.

### Sample size

No formal power calculation was performed, as the study was designed to provide descriptive and exploratory comparisons rather than hypothesis-driven inferential testing. The target sample size was pragmatic and intended to capture a broad range of patient perspectives across prescribing types.

### Data analysis

Likert scale responses were summarised using descriptive statistics, with proportions calculated based on the number of respondents per item to account for missing data. This approach acknowledges potential bias due to non-response, which was assumed to be randomly distributed. For descriptive reporting, response categories were collapsed into ‘agree or strongly agree’ and ‘disagree or strongly disagree’ to aid interpretation; all analyses were conducted using the full 5-point Likert scale.

Differences in patient perspectives by prescribing model were analysed using Chi-square or Fisher’s exact tests, as appropriate. A *p*-value < 0.05 was considered statistically significant. Bonferroni correction was applied (adjusted *p* < 0.0045) to account for multiple pairwise comparisons and to reduce the risk of Type I error. Quantitative analyses were performed using SPSS version 29 (IBM Corporation, Armonk, NY, USA).

Free-text responses were analysed using Braun and Clarke’s 6-step reflexive thematic analysis framework [43]. NVivo version 14.0 (QSR International Pty Ltd., Doncaster, Victoria, Australia) was used to manage and code the data. Two investigators independently familiarised themselves with the data and generated initial codes, which were iteratively refined into themes through team discussion. Reflexive logs were maintained throughout the analysis to ensure ongoing reflection of the content, thematic interpretations, and potential biases.

### Ethics approval

Approval to conduct the study was obtained from the Central Adelaide Local Health Network Human Research Ethics Committee (approval number: 16945, approved: 08/11/2022) and the University of South Australia Human Research Ethics Committee (approval number: 205819, approved: 07/09/2023). All participants provided informed consent prior to participating in the study.

## Results

### Demographics

Two-hundred responses were received (100 collaborative pharmacist prescribing; 100 medical prescribing). Participants were recruited across four hospitals, with the number of respondents varying between sites: Hospital A (n = 72), Hospital B (n = 43), Hospital C (n = 9), Hospital D (n = 72); four participants did not report their hospital. The median age of participants was 72 years, just over half were female, and most lived at home and self-managed their medicines prior to admission. Baseline characteristics did not differ significantly between patients who received collaborative pharmacist prescribing or medical prescribing *(*Table 1*).*

**Table 1:** Participant demographics

	Collaborative pharmacist prescribing(n = 100)	Medical doctor prescribing (n = 100)	*p-*value^1^
*Age (years) median (p25, p75)*	75 (66–82)	71 (64–79)	0.115
Female, n (%)	48 (48%)	58 (58%)	0.397
Male, n (%)	50 (50%)	41 (41%)	
*Residence, n (%)*			
Home alone	28 (28%)	30 (30%)	0.57
Home with family, friends or others	69 (69%)	64 (64%)	
Care facility or retirement village	1 (1%)	1 (1%)	
*Medication management at home, n (%)*			
Self-manages medications	82 (82%)	84 (84%)	0.428
Utilises a dose aid (i.e. Webster-Pak®, dosette box or other weekly or daily medication pack) to manage medications at home	53 (53%)	41 (41%)	0.210

^1^Chi-Square or Fisher’s exact test performed where appropriate

Patients who received collaborative pharmacist prescribing were more likely to report having discussed their medicines with a pharmacist compared with patients who received medical prescribing (86% vs. 56%, *p* < 0.001). There were no significant differences between prescribing types in reported discussions with other healthcare professionals (Table 2).

**Table 2:** Health professionals reported to have discussed medicines with patients

Which health professional/s discussed your medicines with you, n(%)^1^			
	Collaborative pharmacist prescribing (n = 100)	Medical prescribing (n = 100)	*p*-value^2^
Pharmacist	86 (86%)	56 (56%)	< 0.001
Medical doctor	75 (75%)	88 (88%)	0.280
Nurse	33 (33%)	37 (37%)	0.328
Other (please specify)			
Occupational therapist	0	2 (2%)	0.249
Physiotherapist	1(1%)	1 (1%)	0.751
Nobody or unsure	5 (5%)	5 (5%)	0.374

^1^Patients could select multiple response options for this question

^2^Chi-Square or Fisher’s exact test performed where appropriate

### Trust and acceptability of pharmacist prescribing

The majority of patients (> 90%), regardless of the type of prescribing received, agreed or strongly agreed that they trust the pharmacist to ensure they receive the right medicines in hospital and that they trust the pharmacists’ knowledge and training. Just over 70% agreed or strongly agreed that medicines prescribed by pharmacists should be checked by a medical doctor before administration. Most patients (> 98%) disagreed or strongly disagreed that pharmacists should not be involved in collaborative prescribing. There were no statistically significant differences in responses between prescribing types (Table 3).

**Table 3:** Comparison of responses to Likert scale and tick box questions between prescribing groups, n/N (%) respondents to the question

Likert scale questions																		
Question		Prescribing type			Strongly disagree			Disagree			Neutral			Agree			Strongly agree	*p*-value*
*Trust in the healthcare team*																		
I trust the pharmacist to ensure I get the right medicines while I am in hospital		Collaborative pharmacist prescribing			2/100 (2%)^a^			2/100 (2%)^a^			2/100 (2%)^a^			31/100 (31%)^a^			63/100 (63%)^a^	0.371
		Medical prescribing			0/100 (0%)^a^			0/100 (0%)^a^			1/100 (1%)^a^			37/100 (37%)^a^			62/100 (62%)^a^	
I believe that pharmacists have the knowledge and training to write my medicines on the medicine chart		Collaborative pharmacist prescribing			0/98 (0%)^a^			1/98 (1%)^a^			1/98 (1%)^a^			32/98 (33%)^a^			64/98 (65%)^a^	0.706
		Medical prescribing			0/100 (0%)^a^			0/100 (0%)^a^			2/100 (2%)^a^			33/100 (33%)^a^			65/100 (65%)^a^	
I believe that a doctor should check the medicine chart by a pharmacist before the I am given the medicines		Collaborative pharmacist prescribing			4/99 (4%)^a^			12/99 (12%)^a^			13/99 (13%)^a^			37/99 (37%)^a^			33/99 (33%)^a^	0.406
		Medical prescribing			6/100 (6%)^a^			12/100 (12%)^a^			10/100 (10%)^a^			48/100 (48%)^a^			24/100 (24%)^a^	
I do not believe pharmacists should be involved in writing medicines on the medicine chart		Collaborative pharmacist prescribing			62/98 (63%)^a^			33/98 (34%)^a^			1/98 (1%)^a^			1/98 (1%)^a^			1/98 (1%)^a^	0.812
		Medical prescribing			66 (66%)^a^			32/100 (32%)^a^			1/100 (1%)^a^			0/100 (0%)^a^			1/100 (1%)^a^	
*Patient Understanding*																		
My medicine plan was explained in a way that was easy to understand		Collaborative pharmacist prescribing			1/99 (1%)^a^			8/99 (8%)^a^			12/99 (12%)^a^			33/99 (33%)^a^			45/99 (45%)^a^	0.003
		Medical prescribing			0/100 (0%)^a^			10/100 (10%)^a^			6/100 (6%)^a^			55/100 (55%)^b^			25/100 (25%)^b^	
I felt that any questions or concerns I had about my medicines were answered by the prescriber(s)^1^		Collaborative pharmacist prescribing			0/95 (0%)^a^			5/95 (5%)^a^			6/95 (6%)^a^			36/95 (38%)^a^			48/95 (51%)^a^	< 0.001
		Medical prescribing			5/90 (6%)^b^			9/90 (10%)^a^			12/90 (13%)^a^			42/90 (47%)^b^			22/90 (24%)^b^	
*Communication between the healthcare team*																		
There was good communication between the pharmacist and the doctor about my medicines			Collaborative pharmacist prescribing			2/97 (2%)^a^			0/97 (0%)^a^			35/97 (36%)^a^			23/97 (24%)^a^		37/97 (38%)^a^	< 0.001
			Medical prescribing^b^			2/55 (3.5%)^a^			9/55 (16%)^b^			3/55 (5.5%)^b^			27/55 (49%)^a^		14/55 (25%)^b^	
*Involvement in medicine decisions*																		
The prescriber/s^1^ worked with me to plan my medicines in line with my goals and wishes	Collaborative pharmacist prescribing			1/99 (1%)^a^			3/99 (3%)^a^			12/99 (12%)^a^			40/99 (40%)^a^			42/99 (42%)^a^		< 0.001
	Medical prescribing			3/100 (3%)^a^			16/100 (16%)^b^			14/100 (14%)^a^			51/100 (51%)^a^			16/100 (16%)^b^		
I felt that I was encouraged to have a say in decisions about my medicines	Collaborative pharmacist prescribing			1/99 (1%)^a^			9/99 (9%)^a^			10/99 (10%)^a^			38/99 (38%)^a^			41/99 (41%)^a^		0.013
	Medical prescribing			2/98 (2%)^a^			17/98 (17%)^a^			10/98 (10%)^a^			50/98 (51%)^a^			19/98 (19%)^b^		
*Question*	*Prescribing type*			*Not at all involved*			*Somewhat involved*			*Moderately involved*			*Very involved*			*Extremely involved*		
How involved did you feel with the decisions made about your medicines?	Collaborative pharmacist prescribing			9/98 (9%)^a^			13/98 (13%)^a^			17/98 (17%)^a^			28/98 (28.5%)^a^			31/98 (31.5%)^a^		< 0.001
	Medical prescribing			21/100 (21%)^b^			25/100 (25%)^b^			40/100 (40%)^b^			11/100 (11%)^b^			3/100 (3%)^b^		
How involved in decisions made about your medicines would you like to feel	Collaborative pharmacist prescribing			45/99 (45%)^a^			33/99 (33%)^a^			12/99 (12%)^a^			4/99 (4%)^a^			45/99 (45%)^a^		< 0.001
	Medical prescribing			18/100 (18%)^a^			41/100 (41%)^a^			30/100 (30%)^b^			9/100 (9%)^b^			2/100 (2%)^b^		
*Tick box question, n (%)*																		
*Question*	*Prescribing type*			*Yes*^2^			*No*			*p-value*								
Do you have any questions about your medicines which have not been answered	Collaborative pharmacist prescribing			11/100 (11%)			89/100 (89%)			0.011								
	Medical prescribing			6/100 (6%)			94/100 (94%)											

^1^In the survey provided to people who received collaborative pharmacist prescribing, prescriber(s) was referred to as “the team of pharmacist and doctor,” and in the survey for people who received medical prescribing, prescriber(s) was referred to as “the doctor”

^2^This question was only asked of patients who had seen a pharmacist during their admission

*P-value represents overall comparisons between prescribing groups across the full distribution of Likertscale responses using Chi-square or Fisher’s exact tests, as appropriate (*p* < 0.05). Superscript letters indicate post hoc pairwise comparisons of column proportions between groups, with Bonferroniadjustment applied for multiple testing (adjusted *p* < 0.0045). Different superscript letters denotestatistically significant differences between proportions

### Patient understanding

Patients reported high levels of medicines understanding. Over three-quarters of patients agreed or strongly agreed that their medicine plan was explained in a way that was easy to understand*,* with no statistically significant difference observed between patients who received collaborative pharmacist prescribing and those who received medical prescribing. A higher proportion of patients who received collaborative pharmacist prescribing agreed or strongly agreed that any questions or concerns about their medicines were answered compared to those who received medical prescribing (89% vs. 71%, *p* < 0.001). *(*Table 3*).*

In addition, in response to a separate survey item asking whether they had any unanswered questions about their medicines at the time of survey completion, the majority of patients (> 89%) reported that all their questions about medicines had been addressed with no significant difference between prescribing types *(*Table 3*).*

### Interprofessional communication

Fifty-five of the 100 patients who received medical prescribing reported that they saw a pharmacist during their admission, and of those 55 patients, 74% agreed or strongly agreed that there was good communication between the pharmacist and medical doctor about their medicines. This was slightly higher than the proportion of patients who received collaborative pharmacist prescribing who agreed or strongly agreed (60%) with this statement (*p* < 0.001). *(*Table 3*).*

### Patient involvement

Significantly more patients who received collaborative pharmacist prescribing agreed or strongly agreed that the prescriber worked with them to plan their medicines in line with their goals and wishes, compared to patients who received medical prescribing (82% vs. 67%, *p* < 0.001).

When asked about their level of involvement in these decisions, a higher proportion of patients in the collaborative pharmacist prescribing group felt very or extremely involved (60% vs. 14%, *p* < 0.001). Desired levels of involvement did not differ between groups *(*Table 3*).*

### Qualitative analysis

Analysis of free text responses identified four key themes reflecting participant’s perceptions of pharmacist prescribing: (1) accessibility to pharmacists and efficiency of care, (2) interprofessional collaboration and error reduction, (3) patients’ awareness of medicines and shared decision-making, and (4) confidence in pharmacists’ knowledge and satisfaction in care.

#### Theme 1: accessibility to pharmacists and efficiency of care

Patients perceived increased accessibility to pharmacists through collaborative pharmacist prescribing, particularly in relation to opportunities to ask questions, receive explanations, and engage in discussions about their medicines. Patients described pharmacists as approachable, available, and able to spend time addressing medicine-related concerns.

*“It gives me the opportunity to ask questions about meds if the doctor is not available.”* (Collaborative pharmacist prescribing patient #1)

*“They [the pharmacist] listened to me. They spent time answering my questions. They are good at communicating in a way that I understand.”* (Collaborative pharmacist prescribing patient #5).

In addition, patients perceived that collaborative prescribing may improve efficiency of care by enabling more effective use of available healthcare resources. Participants described perceived improvements in the timeliness of care and more efficient allocation of medical staff time with collaborative pharmacist prescribing.

*“It sounds like it might make the process quicker and give the doctors time to do other things. I am surprised it hasn’t been done sooner.”* (Medical prescribing patient #51).

*“Frees up Dr’s (sic) time.”* (Collaborative pharmacist prescribing patient #2).

Patients also perceived that increased pharmacist involvement earlier in the prescribing process could improve efficiency related to medication supply and discharge processes, potentially reducing delays and multiple medication changes.

*“…My medicines have been changed multiple times. It might have been quicker and less changes if the pharmacist was involved from the get go.”* (Medical prescribing patient #35).

*“More thorough, less of a wait in getting my medicines if pharmacists are involved.”* (Collaborative pharmacist prescribing patient #98)

#### Theme 2: interprofessional collaboration and reduction in errors

Patients consistently described interprofessional collaboration between pharmacists and medical doctors as enhancing their sense of safety and reassurance. Many perceived that close communication between the healthcare professionals reduced medication errors and improved coordination of care.

*“It would give me more confidence knowing they are working together, would avoid mistakes I would imagine.”* (Medical prescribing patient #36).

*“They [pharmacist and doctor] are highly trained and knowledgeable. Reassuring knowing they worked as a team. I’m sure there are less errors that way.”* (Collaborative pharmacist prescribing patient #5).

A patient drew on prior hospital experience to contextualise this perception, suggesting that collaborative pharmacist prescribing was associated with fewer medication errors compared to previous admissions.

*“Based on previous admissions to now there have already been less medication errors...”* (Collaborative pharmacist prescribing patient #1).

Many patients perceived benefits of bringing together the different yet complementary expertise of pharmacists and medical doctors in the prescribing process:

*“Two people checking from the start, both experts. It’d be better for us as patients.”* (Medical prescribing patient #26).

*“I think it’s a good idea as doctors are very specific but pharmacists are generalists.”* (Collaborative pharmacist prescribing patient #6).

#### Theme 3: patients’ awareness of medicines and shared decision-making

Patients viewed collaborative pharmacist prescribing as an opportunity to improve their understanding and awareness of medicines, which in turn enhanced their sense of empowerment, participation in treatment decisions, and a sense of reassurance about their care. Patients who received collaborative pharmacist prescribing appreciated being more informed about their medicines. A patient perceived that the benefits experienced during the admission would extend beyond hospital, fostering confidence in medicine management post-discharge.

*“I will be more aware of the medicines I am taking in hospital, being informed of side-effects, being reassured I am not prescribed anything that I am allergic to.”* (Medical prescribing patient #2).

*“I felt more included. I was more informed about changes, and I feel good about my medicines in here and when I go home.”* (Collaborative pharmacist prescribing patient #1).

#### Theme 4: confidence in pharmacists’ knowledge and satisfaction in care

Patients expressed confidence in pharmacists’ medicines knowledge, which contributed to overall satisfaction with care. This confidence appeared to reflect patients’ perceptions of pharmacists’ medicines expertise and their ability to clearly communicate medicine-related information, particularly around benefits, risks, and potential interactions. This in turn, fostered a sense of reassurance and safety in medication management, with pharmacist involvement perceived as an additional safeguard. For some patients, this confidence extended beyond the immediate hospital stay, contributing to greater trust in their ongoing treatment more broadly.

*“They [the pharmacist] may be more open to discussion and can explain the benefits and side effects better and off the top of their heads. I’d feel more comfortable taking a new medicine that way.”* (Medical prescribing patient #77)

*“Having a pharmacist involved confirms that we don’t have any medicines that clash with each other and that I am currently taking what I am supposed to be taking.”* (Collaborative pharmacist prescribing patient #38).

## Discussion

### Summary of findings

Patients in our study reported trust in pharmacists’ medicines expertise and confidence in pharmacists’ ability to prescribe within a collaborative model. Quantitative results demonstrated that patients who received collaborative pharmacist prescribing reported significantly greater agreement that questions or concerns about their medicines were addressed, compared with patients who received medical prescribing alone. While free-text responses provided contextual insight, the primary findings of this study are supported by statistically significant differences observed in patient-reported measures of enhanced involvement in shared decision-making.

Together, these findings suggest that collaborative pharmacist prescribing may enhance key aspects of the patient experience, particularly shared-decision making and reassurance about medication management during hospital admission. Importantly, patients supported pharmacist prescribing when embedded within a multi-disciplinary framework. The majority expressed a preference for medical oversight of pharmacist prescribing, highlighting the importance of interprofessional collaboration and shared accountability in hospital settings.

### Comparison with literature

Patients in our study expressed trust in pharmacists’ knowledge and prescribing ability, regardless of whether they received pharmacist prescribing. These findings are consistent with previous studies reporting positive patient perceptions of pharmacist competence and trust in prescribing roles [19]. This aligns with patient-centred medication safety frameworks, including P-MEDS and Donabedian’s Quality of Care model, which conceptualise professional competence and effective communication as key processes underpinning safe and high-quality care [37, 38].

Most participants supported a collaborative model of pharmacist prescribing and expressed preferences for medical doctors checking medicines prescribed by a pharmacist. This preference likely reflects an expectation of shared responsibility and aligns with the design of our collaborative model where pharmacists work with medical doctors to create and agree upon the medicine plan before prescribing. Similarly, other studies have reported patient preferences for pharmacist prescribing within defined, collaborative boundaries [19, 44]. While the findings of the present study highlight the acceptability of collaborative pharmacist prescribing in hospital inpatient care, emerging evidence, particularly from primary care contexts, suggest growing support for pharmacist-led prescribing models [45–47]. However, emerging literature, particularly from primary care settings suggests growing patient acceptance of independent prescribing models [45–47]. Together these findings suggest that patient acceptability of pharmacy prescribing may be context dependent, with collaboration and oversight remaining important in multi-disciplinary hospital settings.

Evidence from hospital inpatient settings has focused predominantly on clinical, safety, and system-level outcomes rather than patient perspectives and experiences. This relative scarcitymay reflect the realities of acute hospital care, where prescribing decisions often occur during periods of acute illness, time pressure, and short hospital admissions, limiting opportunities for patient engagement and for the collection of patient-reported outcomes. Within this context, the present study provides novel insights into patients’ perceptions and experiences of collaborative pharmacist prescribing during inpatient care, addressing an important gap in the literature and complementing existing inpatient evaluations on clinical and system-level outcomes.

Regardless of who prescribed their medicines, participants emphasised the importance of collaboration between pharmacists and medical doctors in supporting safe medication management during their admission. Patients described pharmacists and medical doctors as having distinct yet complementary roles, and highlighted effective communication and coordination between healthcare professionals as central to reducing medication errors. These perceptions align with the Donabedian framework, which recognises communication and coordination as key process elements of quality care [37]. Interprofessional teamwork is also a core component of patient-centred medication safety within the P-MEDs framework [38]. Such collaboration supports shared decision-making, strengthens patient confidence, and enhances patients’ trust in care delivery [48–51].

Despite the recognised importance of interprofessional collaboration, pharmacists’ roles within multidisciplinary healthcare teams have traditionally been characterised by reactive communication, whereby medication issues are identified and addressed after they arise [51–53]. For example, pharmacists often review medicines after they have been prescribed, and communicate any identified issues to the medical team, in many cases after the medicines have already been administered to the patient [51–53]. This reactive approach, has been associated with incomplete or delayed information transfer, increasing the risk of medication error and subsequent patient harm [52]. By shifting pharmacists from a reactive to a proactive role in medicine management, collaborative pharmacist prescribing facilitates earlier pharmacist involvement in prescribing decisions. A 2021 systematic review and meta-analysis demonstrated that pharmacist involvement within multidisciplinary teams is associated with improved outcomes, including reduced hospital readmissions, improvements in quality of life and improved cost-effectiveness [54]. While the clinical outcomes of collaborative pharmacist prescribing are well established [7–12], its impact on patient experiences in the inpatient hospital setting is less understood. Our qualitative findings reveal that patients associate collaborative pharmacist prescribing with improvements in safety, reinforcing quantitative findings of increased satisfaction and partnership in care. Patients also perceived improved access to pharmacists with the pharmacist prescribing model, noting potential flow-on benefits for medical staff. Similar perceptions have been reported previously among both patients and pharmacists [19].

### Strengths and limitations

A key strength of this study is the use of established conceptual frameworks in healthcare quality and patient safety, with the Donabedian model and P-MEDS framework informing questionnaire development and interpretation of medication-related care processes and patient-reported experiences [37, 38]. The structured survey captured patient experiences across relevant domains, while free-text responses provided additional context to support the interpretation of findings.

Participants in the medical prescribing group were approached from among random eligible inpatients within the same clinical units where collaborative pharmacist prescribing was implemented; however, no formal matching or stratification by patient characteristics was undertaken. At the study sites, pharmacists commonly prioritise patients with complex medication needs for pharmacist prescribing due to staffing constraints. This means that although baseline characteristics between groups were similar, residual confounding or selection bias due to unmeasured differences in clinical complexity of patients cannot be ruled out. The study was not specially funded to recruit a translator for research purposes. However, where accredited translators were present as part of usual clinical care, or if a family member or friend was present and capable of translating, then they were permitted to assist patients during questionnaire completion to enable equitable participation of patients from non-English speaking backgrounds. The number of participants requiring interpreter support was not recorded. Other limitations include the inability to record the number of eligible patients approached for participation, and the potential for social desirability bias in responses [55]. To minimise bias, pharmacist prescribers were not present during survey completion and did not collect completed surveys. Finally, recruitment across multiple hospitals with different prescribers represents a strength, enhancing the diversity of the clinical contexts represented and supporting the generalisability of the findings, despite unequal recruitment across sites.

## Conclusion

Patients reported high trust in pharmacists’ knowledge and collaborative prescribing ability, regardless of whether they received pharmacist or medical prescribing. Patients who experienced collaborative pharmacist prescribing reported a greater level of involvement in shared-decision making related to prescribing compared to patients who received medical prescribing alone. These findings were further supported by patients’ free-text responses, which highlighted enhanced safety due to collaboration, and a deeper understanding of their medicines. Collectively, these findings reinforce the value of embedding pharmacists in collaborative prescribing roles to enhance patient-centered care, support shared decision-making, and contribute to better health outcomes. As collaborative prescribing models continue to expand in hospital settings, incorporating patient perspectives alongside clinical and system-level outcomes will be essential to ensure these services remain patient-centered, acceptable and reflective of the complexities of care delivery in acute hospital settings.

## Supplementary Information

Below is the link to the electronic supplementary material.

Supplementary file1 (PDF 417 KB)

## Data Availability

The data that support the findings of this study are not publicly available due to ethical and privacy restrictions involving patient confidentiality. De-identified data may be made available from the corresponding author (HA) upon reasonable request and with approval from the appropriate institutional ethics committee.
