# Supplementary material for: Patient perspectives and experiences of collaborative pharmacist prescribing in the hospital inpatient setting: a cross-sectional survey
Source: Int J Clin Pharm. 2026 Jun 27;48(5):1991–2004. doi: 10.1007/s11096-026-02178-0 (PMC13569529; doi:10.1007/s11096-026-02178-0)
Supplement: Supplementary file 1 [file 11096_2026_2178_MOESM1_ESM.pdf]

Patient perspectives and experiences of collaborative pharmacist prescribing in the hospital inpatient setting: a cross-sectional survey

-- Supplementary Files --

Hana Amer[1,2,3] https://orcid.org/0000-0002-0248-7241, Sally Marotti[1,2,4] https://orcid.org/0000-0003-3098-6096, Jacinta Johnson[1,2] https://orcid.org/0000-0003-4786-022X , Imaina Widagdo[1] https://orcid.org/0000-0002-0669-0183, Kirsten Burns[1,2] https://orcid.org/0009-0002-7938-3819, Sharon Goldsworthy[2], Ramim Hossin[1,2] https://orcid.org/0009-0008-5623-9711, Molly Astley [1,2], Lisa Kalisch Ellett[1] https://orcid.org/0000-0001-5063-6128.

[1]Quality Use of Medicines and Pharmacy Research Centre, School of Pharmacy and Biomedical Sciences, College of Health, Adelaide University, South Australia 5001
[2]SA Pharmacy, SA Health, L7 11 Hindmarsh Square, Adelaide, South Australia 5000
[3]Faculty of Medicine and Health, School of Pharmacy, The University of Sydney, New South Wales 2050
[4]College of Medicine and Public Health, Flinders University

Corresponding author details
Hana Amer
Quality Use of Medicines and Pharmacy Research Centre, School of Pharmacy and Biomedical Sciences, College of Health, Adelaide University, South Australia 5001
hana.amer@adelaide.edu.au
https://orcid.org/0000-0002-0248-7241

Supplementary File 1. STROBE checklist for cross-sectional studies

STROBE Statement—Checklist of items that should be included in reports of ***cross-sectional studies***

	Item No	Recommendation	Page No
**Title and abstract**	1	(*a*) Indicate the study's design with a commonly used term in the title or the abstract	#1
		(*b*) Provide in the abstract an informative and balanced summary of what was done and what was found	#2
**Introduction**			
Background/rationale	2	Explain the scientific background and rationale for the investigation being reported	#4
Objectives	3	State specific objectives, including any prespecified hypotheses	#5
**Methods**			
Study design	4	Present key elements of study design early in the paper	#6
Setting	5	Describe the setting, locations, and relevant dates, including periods of recruitment, exposure, follow-up, and data collection	#6
Participants	6	(*a*) Give the eligibility criteria, and the sources and methods of selection of participants	#6
Variables	7	Clearly define all outcomes, exposures, predictors, potential confounders, and effect modifiers. Give diagnostic criteria, if applicable	#7
Data sources/ measurement	8*	For each variable of interest, give sources of data and details of methods of assessment (measurement). Describe comparability of assessment methods if there is more than one group	#7
Bias	9	Describe any efforts to address potential sources of bias	#7
Study size	10	Explain how the study size was arrived at	#7
Quantitative variables	11	Explain how quantitative variables were handled in the analyses. If applicable, describe which groupings were chosen and why	#7

Statistical methods	12	(*a*) Describe all statistical methods, including those used to control for confounding	#7
		(*b*) Describe any methods used to examine subgroups and interactions	#7
		(*c*) Explain how missing data were addressed	#7
		(*d*) If applicable, describe analytical methods taking account of sampling strategy	N/A
		(*e*) Describe any sensitivity analyses	N/A
**Results**			
Participants	13*	(a) Report numbers of individuals at each stage of study—eg numbers potentially eligible, examined for eligibility, confirmed eligible, included in the study, completing follow-up, and analysed	#9<br>Proportions were reported based on the number of respondents per item to account for missing data.
		(b) Give reasons for non-participation at each stage	N/A
		(c) Consider use of a flow diagram	N/A
Descriptive data	14*	(a) Give characteristics of study participants (eg demographic, clinical, social) and information on exposures and potential confounders	#9
		(b) Indicate number of participants with missing data for each variable of interest	Proportions were reported based on the number of respondents per item to account for missing data.
Outcome data	15*	Report numbers of outcome events or summary measures	#9-13
Main results	16	(*a*) Give unadjusted estimates and, if applicable, confounder-adjusted estimates and their precision (eg, 95% confidence interval). Make clear which confounders were adjusted for and why they were included	#9-13
		(*b*) Report category boundaries when continuous variables were categorized	N/A
		(*c*) If relevant, consider translating estimates of relative risk into absolute risk for a meaningful time period	N/A

Other analyses	17	Report other analyses done—eg analyses of subgroups and interactions, and sensitivity analyses	N/A
**Discussion**			
Key results	18	Summarise key results with reference to study objectives	#17
Limitations	19	Discuss limitations of the study, taking into account sources of potential bias or imprecision. Discuss both direction and magnitude of any potential bias	#18
Interpretation	20	Give a cautious overall interpretation of results considering objectives, limitations, multiplicity of analyses, results from similar studies, and other relevant evidence	#18
Generalisability	21	Discuss the generalisability (external validity) of the study results	#18
**Other information**			
Funding	22	Give the source of funding and the role of the funders for the present study and, if applicable, for the original study on which the present article is based	#24

*Give information separately for exposed and unexposed groups.

Note: An Explanation and Elaboration article discusses each checklist item and gives methodological background and published examples of transparent reporting. The STROBE checklist is best used in conjunction with this article (freely available on the Web sites of PLoS Medicine at http://www.plosmedicine.org/, Annals of Internal Medicine at http://www.annals.org/, and Epidemiology at http://www.epidem.com/). Information on the STROBE Initiative is available at www.strobe-statement.org.

Supplementary File 2. A Consensus-Based Checklist for Reporting Of Survey Studies (CROSS)

Section/topic	Item	Item description	Reported on page #
**Title and abstract**			
Title and abstract	1a	State the word “survey” along with a commonly used term in title or abstract to introduce the study’s design.	#1-2
	1b	Provide an informative summary in the abstract, covering background, objectives, methods, findings/results, interpretation/discussion, and conclusions.	#2
**Introduction**			
Background	2	Provide a background about the rationale of study, what has been previously done, and why this survey is needed.	#4
Purpose/aim	3	Identify specific purposes, aims, goals, or objectives of the study.	#5
**Methods**			
Study design	4	Specify the study design in the methods section with a commonly used term (e.g., cross-sectional or longitudinal).	#6
Data collection methods	5a	Describe the questionnaire (e.g., number of sections, number of questions, number and names of instruments used).	#6
	5b	Describe all questionnaire instruments that were used in the survey to measure particular concepts. Report target population, reported validity and reliability information, scoring/classification procedure, and reference links (if any).	#6
	5c	Provide information on pretesting of the questionnaire, if performed (in the article or in an online supplement). Report the method of pretesting, number of times questionnaire was pre-tested, number and demographics of participants used for pretesting, and the level of similarity of demographics between pre-testing participants and sample population.	#7
	5d	Questionnaire if possible, should be fully provided (in the article, or as appendices or as an online supplement).	Supplementary File 3
Sample characteristics	6a	Describe the study population (i.e., background, locations, eligibility criteria for participant inclusion in survey, exclusion criteria).	#6

	6b	Describe the sampling techniques used (e.g., single stage or multistage sampling, simple random sampling, stratified sampling, cluster sampling, convenience sampling). Specify the locations of sample participants whenever clustered sampling was applied.	#7
	6c	Provide information on sample size, along with details of sample size calculation.	#7
	6d	Describe how representative the sample is of the study population (or target population if possible), particularly for population-based surveys.	#7
Survey administration	7a	Provide information on modes of questionnaire administration, including the type and number of contacts, the location where the survey was conducted (e.g., outpatient room or by use of online tools, such as SurveyMonkey).	#7
	7b	Provide information of survey's time frame, such as periods of recruitment, exposure, and follow-up days.	#7
	7c	Provide information on the entry process:<br>–>For non-web-based surveys, provide approaches to minimize human error in data entry.<br>–>For web-based surveys, provide approaches to prevent "multiple participation" of participants.	#7
Study preparation	8	Describe any preparation process before conducting the survey (e.g., interviewers' training process, advertising the survey).	#6
Ethical considerations	9a	Provide information on ethical approval for the survey if obtained, including informed consent, institutional review board [IRB] approval, Helsinki declaration, and good clinical practice [GCP] declaration (as appropriate).	#8
	9b	Provide information about survey anonymity and confidentiality and describe what mechanisms were used to protect unauthorized access.	#6-7
Statistical analysis	10a	Describe statistical methods and analytical approach. Report the statistical software that was used for data analysis.	#7
	10b	Report any modification of variables used in the analysis, along with reference (if available).	N/A
	10c	Report details about how missing data was handled. Include rate of missing items, missing data mechanism (i.e., missing completely at random [MCAR], missing at random [MAR] or missing not at random [MNAR]) and methods	#7

		used to deal with missing data (e.g., multiple imputation).	
	10d	State how non-response error was addressed.	#7
	10e	For longitudinal surveys, state how loss to follow-up was addressed.	N/A
	10f	Indicate whether any methods such as weighting of items or propensity scores have been used to adjust for non-representativeness of the sample.	N/A
	10g	Describe any sensitivity analysis conducted.	N/A
**Results**			
Respondent characteristics	11a	Report numbers of individuals at each stage of the study. Consider using a flow diagram, if possible.	#9
	11b	Provide reasons for non-participation at each stage, if possible.	N/A
	11c	Report response rate, present the definition of response rate or the formula used to calculate response rate.	#9
	11d	Provide information to define how unique visitors are determined. Report number of unique visitors along with relevant proportions (e.g., view proportion, participation proportion, completion proportion).	#7
Descriptive results	12	Provide characteristics of study participants, as well as information on potential confounders and assessed outcomes.	#9
Main findings	13a	Give unadjusted estimates and, if applicable, confounder-adjusted estimates along with 95% confidence intervals and p-values.	#9-13
	13b	For multivariable analysis, provide information on the model building process, model fit statistics, and model assumptions (as appropriate).	N/A
	13c	Provide details about any sensitivity analysis performed. If there are considerable amount of missing data, report sensitivity analyses comparing the results of complete cases with that of the imputed dataset (if possible).	N/A
**Discussion**			
Limitations	14	Discuss the limitations of the study, considering sources of potential biases and imprecisions, such as non-representativeness of sample, study design, important uncontrolled confounders.	#18

Interpretations	15	Give a cautious overall interpretation of results, based on potential biases and imprecisions and suggest areas for future research.	#17-19
Generalizability	16	Discuss the external validity of the results.	#18
**Other sections**			
Role of funding source	17	State whether any funding organization has had any roles in the survey's design, implementation, and analysis.	#24
Conflict of interest	18	Declare any potential conflict of interest.	#24
Acknowledgements	19	Provide names of organizations/persons that are acknowledged along with their contribution to the research.	#24

Sharma A, Minh Duc NT, Luu Lam Thang T, Nam NH, Ng SJ, Abbas KS, Huy NT, Marušić A, Paul CL, Kwok J, Karbwang J, de Waure C, Drummond FJ, Kizawa Y, Taal E, Vermeulen J, Lee GHM, Gyedu A, To KG, Verra ML, Jacqz-Aigrain ÉM, Leclercq WKG, Salminen ST, Sherbourne CD, Mintzes B, Lozano S, Tran US, Matsui M, Karamouzian M. A Consensus-Based Checklist for Reporting of Survey Studies (CROSS). J Gen Intern Med. 2021 Oct;36(10):3179-3187. doi: 10.1007/s11606-021-06737-1. Epub 2021 Apr 22. PMID: 33886027; PMCID: PMC8481359.

Supplementary File 3. Comparison of collaborative pharmacist prescribing and medical prescribing surveys

Two versions of the questionnaire were developed, one for patients who received collaborative pharmacist prescribing and one for patients who received medical prescribing. Questionnaires were largely identical, with minor differences in wording and introductory text to reflect the prescribing model received.

Supplementary File 3 below provides both versions of the survey side-by-side to facilitate comparison of corresponding items. Survey questions are not presented in chronological order but are instead grouped by question type (tick-box, likert Scale and short-answer questions).

**Collaborative pharmacist prescribing survey**	**Medical prescribing survey**
**About you**	**About you**
Which hospital are you in?*<br><br>☐ Hospital A<br>☐ Hospital B<br>☐ Hospital C<br>☐ Hospital D	Which hospital are you in? *<br><br>☐ Hospital A<br>☐ Hospital B<br>☐ Hospital C<br>☐ Hospital D
How old are you (in years)?<br><br>______	How old are you (in years)?<br><br>______
What gender are you?<br><br>☐ Male<br>☐ Female<br>☐ Other (please specify) ______<br>☐ Prefer not to say	What gender are you?<br><br>☐ Male<br>☐ Female<br>☐ Other (please specify) ______<br>☐ Prefer not to say
Are you of Aboriginal and/or Torres Strait Islander origin?<br><br>☐ No<br>☐ Yes, Aboriginal<br>☐ Yes, Torres Strait Islander<br>☐ Yes, both Aboriginal and Torres Strait Islander	Are you of Aboriginal and/or Torres Strait Islander origin?<br><br>☐ No<br>☐ Yes, Aboriginal<br>☐ Yes, Torres Strait Islander<br>☐ Yes, both Aboriginal and Torres Strait Islander
How do you live?<br><br>☐ I live at home alone<br>☐ I live at home with family, friends or others<br>☐ I live in a care facility (e.g. nursing home)<br>☐ Other (please specify) _____________	How do you live?<br><br>☐ I live at home alone<br>☐ I live at home with family, friends or others<br>☐ I live in a care facility (e.g. nursing home)<br>☐ Other (please specify) _____________
Did you look after your own medicines before coming into hospital?<br><br>☐ Yes<br>☐ No, someone helps me with them	Did you look after your own medicines before coming into hospital?<br><br>☐ Yes<br>☐ No, someone helps me with them

Did you use a dose aid (Webster-Pak®, dosette box or other weekly or daily medicine pack) to help you manage your medicines before you came into hospital?<br>☐ Yes<br>☐ No, I take the medicines directly out of their packets	Did you use a dose aid (Webster-Pak®, dosette box or other weekly or daily medicine pack) to help you manage your medicines before you came into hospital?<br>☐ Yes<br>☐ No, I take the medicines directly out of their packets
How much do you agree with the following?<br>I am confident with managing my medicines at home (if applicable)	How much do you agree with the following?<br>I am confident with managing my medicines at home (if applicable)
**Tick-box questions**	**Tick-box questions**
1) Which health professionals (if you know) discussed your medicines with you. (Select all that apply):<br>☐ Doctor<br>☐ Pharmacist<br>☐ Nurse<br>☐ Other (Please specify) ______<br>☐ Unsure	1) Which health professionals (if you know) discussed your medicines with you. (Tick all that apply):<br>☐ Doctor<br>☐ Pharmacist<br>☐ Nurse<br>☐ Other (Please specify) ______<br>☐ Unsure
**Likert Scale Questions**	**Likert Scale Questions**
**How much do you agree with the following statements?**<br>(5-point Likert scale: Strongly Agree, Agree, Neutral, Disagree, Strongly Disagree)	**How much do you agree with the following statements?**<br>(5-point Likert scale: Strongly Agree, Agree, Neutral, Disagree, Strongly Disagree)
2) I trust the pharmacist to ensure I get the right medicines while I am in hospital	2) I trust the doctor to ensure I get the right medicines when I am in hospital<br>15) I trust a pharmacist to ensure I get the right medicines when I am in hospital
3) The pharmacist and doctor worked with me to plan my medicines in hospital, in line with my goals and wishes	3) The doctor worked with me to plan my medicines in hospital, in line with my goals and wishes
6) My medicine plan was explained to me in a way that was easy to understand	6) My medicine plan was explained to me in a way that was easy to understand
7) I felt that any questions or concerns I had about my medicines were answered by the team	7) I felt that any questions or concerns I had about my medicines was answered by the doctors
8) There was good communication between the pharmacist and doctor about my medicines	10) Have you seen a pharmacist during your time in hospital?<br>☐ Yes<br>☐ No or not sure

	If yes:<br><br>11) There was good communication between the pharmacist and doctor about my medicines<br><br>12) I felt that any questions or concerns I had about my medicines were answered by the pharmacist
15) I do not believe pharmacists should be involved in writing my medicines on the medicine chart	18) I do not believe pharmacists should be involved in writing medicines on the medicine chart
**How much do you agree with the following statements?**<br>(5-point Likert scale: Not at all involved, Somewhat involved, moderately involved, Very involved, Extremely involved)	**How much do you agree with the following statements?**<br>(5-point Likert scale: Not at all involved, Somewhat involved, Moderately involved, Very involved, Extremely involved)
4) How involved did you feel with the decisions made about your medicines?	4) How involved did you feel with the decisions made about your medicines?
5) How involved in decisions about your medicines would you like to feel?	5) How involved in decisions about your medicines would you like to feel?
**Short answer questions (free-text box)**	**Short answer questions (free-text box)**
9) Do you have any questions about your medicines which have not been answered? If yes, please list.	8) Do you have any questions about your medicines which have not been answered? If yes, please list.
10) What do you think are the benefits of having a pharmacist write your medicines on the medicine chart?	Some patients are having their medicines written on the medicine chart by pharmacists after the pharmacist and the doctor have worked together to understand the patient's needs and goals and worked out what medicines are needed to best care for the patient.<br><br>The following questions are relating to your opinions on this new way of providing care.<br><br>13) What do you think are the benefits of having a pharmacist write up your medicines on the medicine chart?
11) Are there any reasons why a pharmacist should not write your medicines on the medicine chart? Please list.	14) Are there any reasons why a pharmacist should not write your medicines on the medicine chart? Please list.

*The names of the individual hospital sites have been redacted to maintain confidentiality

Supplementary File 4. Survey instruments

Collaborative Pharmacist Prescribing Survey

<u>About you</u>
Which hospital are you in? (Multiple choice – single answer)

- ☐ Hospital A
- ☐ Hospital B
- ☐ Hospital C
- ☐ Hospital D

How old are you (in years)?

____

What gender are you? (Multiple choice – single answer)

- ☐ Male
- ☐ Female
- ☐ Other (please specify) _______
- ☐ Prefer not to say

Are you of Aboriginal and/or Torres Strait Islander origin? (Multiple choice – single answer)

- ☐ No
- ☐ Yes, Aboriginal
- ☐ Yes, Torres Strait Islander
- ☐ Yes, both Aboriginal and Torres Strait Islander

How do you live? (Multiple choice – single answer)

- ☐ I live at home alone
- ☐ I live at home with family, friends or others
- ☐ I live in a care facility (e.g. nursing home)
- ☐ Other (please specify) ______________

Did you look after your own medicines before coming into hospital? (Multiple choice – single answer)

- ☐ Yes
- ☐ No, someone helps me with them

Did you use a dose aid (Webster-Pak®, dosette box or other weekly or daily medication pack) to help you manage your medicines before you came into hospital? (Multiple choice – single answer, on Qualtrics this question was only displayed to patients to answered yes to "Did you look after your own medicines before coming into hospital?")

- ☐ Yes
- ☐ No, I take the medicines directly out of their packets

How much do you agree with the following?
5-point Likert scale: Strongly Agree, Agree, Neutral, Disagree, Strongly Disagree

I am confident with managing my medicines at home (if applicable)
(On Qualtrics this question was only displayed to patients to answered yes to "Did you look after your own medicines before coming into hospital?")

Strongly agree	Agree	Neutral	Disagree	Strongly disagree
1	2	3	4	5

A Pharmacist spoke to you when you came into hospital. After this your doctor and pharmacist worked together to plan your medicines in hospital. Your pharmacist then wrote your medicines on the medicine chart according to this plan (also known as medicine charting). This is different to the normal process of the doctor charting the medicines.

The following questions are related to this recent experience

1)Which health professionals (if you know) discussed your medicines with you. (Select all that apply):

- ☐ Doctor
- ☐ Pharmacist
- ☐ Nurse
- ☐ Other (Please specify) ______
- ☐ Unsure

How much do you agree with the following?
5-point Likert scale: Strongly Agree, Agree, Neutral, Disagree, Strongly Disagree

2) I trust the pharmacist to ensure I get the right medicines while I am in hospital

Strongly agree	Agree	Neutral	Disagree	Strongly disagree
1	2	3	4	5

3) The <u>pharmacist and doctor</u> worked with me to plan my medicines in line with my goals and wishes

Strongly agree	Agree	Neutral	Disagree	Strongly disagree
1	2	3	4	5

5-point Likert scale: Not at all involved, Somewhat involved, Moderately involved, Very involved, Extremely involved

4) How involved <u>did you feel</u> with the decisions made about your medicines?

Not all involved	Slightly involved	Moderately involved	Very involved	Extremely involved
1	2	3	4	5

5) How involved in decisions about your medicines would you <u>like to feel</u>?

Not all involved	Slightly involved	Moderately involved	Very involved	Extremely involved
1	2	3	4	5

5-point Likert scale: Strongly Agree, Agree, Neutral, Disagree, Strongly Disagree

6) My medicine plan was explained to me in a way that was easy to understand

Strongly agree	Agree	Neutral	Disagree	Strongly disagree
1	2	3	4	5

7) I felt that any questions or concerns I had about my medicines were answered by <u>the team</u>

Strongly agree	Agree	Neutral	Disagree	Strongly disagree
1	2	3	4	5

8) There was good communication between the pharmacist and doctor about my medicines

Strongly agree	Agree	Neutral	Disagree	Strongly disagree	Unsure
1	2	3	4	5	6

Free-text box questions

9) Do you have any questions about your medicines which have not been answered? If yes, please list.

10) What do you think are the benefits of having a pharmacist write your medicines on the medicine chart? Please list.

11) Are there any reasons why a pharmacist should not write your medicines on the medicine chart? Please list.

How much do you agree with the following?
5-point Likert scale: Strongly Agree, Agree, Neutral, Disagree, Strongly Disagree

12) I feel that I was encouraged to have a say in decisions about my medicines

Strongly agree	Agree	Neutral	Disagree	Strongly disagree
1	2	3	4	5

13) I believe that pharmacists have the knowledge and training to write my medicines on the medicine chart

Strongly agree	Agree	Neutral	Disagree	Strongly disagree
1	2	3	4	5

14) I believe that a doctor should check the medicine chart written by a pharmacist before I am given the medicines

Strongly agree	Agree	Neutral	Disagree	Strongly disagree
1	2	3	4	5

15) I do not believe pharmacists should be involved in writing my medicines on the medicine chart

Strongly agree	Agree	Neutral	Disagree	Strongly disagree
1	2	3	4	5

Medical Prescribing Survey

<u>About you</u>

Which hospital are you in? (Multiple choice – single answer)

- ☐ Hospital A
- ☐ Hospital B
- ☐ Hospital C
- ☐ Hospital D

How old are you (in years)?

____

What gender are you? (Multiple choice – single answer)

- ☐ Male
- ☐ Female
- ☐ Other (please specify) ______
- ☐ Prefer not to say

Are you of Aboriginal and/or Torres Strait Islander origin? (Multiple choice – single answer)

- ☐ No
- ☐ Yes, Aboriginal
- ☐ Yes, Torres Strait Islander
- ☐ Yes, both Aboriginal and Torres Strait Islander

How do you live? (Multiple choice – single answer)

- ☐ I live at home alone
- ☐ I live at home with family, friends or others
- ☐ I live in a care facility (e.g. nursing home)
- ☐ Other (please specify) _____________

Did you look after your own medicines before coming into hospital? (Multiple choice – single answer)

- ☐ Yes
- ☐ No, someone helps me with them

Did you use a dose aid (Webster-Pak®, dosette box or other weekly or daily medication pack) to help you manage your medicines before you came into hospital? (Multiple choice – single answer, on Qualtrics this question was only displayed to patients to answered yes to "Did you look after your own medicines before coming into hospital?")

- ☐ Yes
- ☐ No, I take the medicines directly out of their packets

How much do you agree with the following?
5-point Likert scale: Strongly Agree, Agree, Neutral, Disagree, Strongly Disagree

I am confident with managing my medicines at home (if applicable)
(On Qualtrics this question was only displayed to patients to answered yes to "Did you look after your own medicines before coming into hospital?")

Strongly agree	Agree	Neutral	Disagree	Strongly disagree
1	2	3	4	5

1) **Which health professionals (if you know) discussed your medicines with you.** (Tick all that apply):
    - ☐ Doctor
    - ☐ Pharmacist
    - ☐ Nurse
    - ☐ Other (Please specify) _____

☐ Unsure

How much do you agree with the following?

2) I trust the doctor to ensure I get the right medicines when I am in hospital

Strongly agree	Agree	Neutral	Disagree	Strongly disagree
1	2	3	4	5

3) The doctor worked with me to plan my medicines in hospital, in line with my goals and wishes

Strongly agree	Agree	Neutral	Disagree	Strongly disagree
1	2	3	4	5

4) How involved did you feel with the decisions made about your medicines??

Not involved at all	Slightly involved	Moderately involved	Very involved	Extremely involved
1	2	3	4	5

5) How involved would you like to feel with the decisions made about your medicines?

Not involved at all	Slightly involved	Moderately involved	Very involved	Extremely involved
1	2	3	4	5

6) My medicine plan was explained to me in a way that was easy to understand

Strongly agree	Agree	Neutral	Disagree	Strongly disagree
1	2	3	4	5

7) I felt that any questions or concerns I had about my medicines was answered by the doctors

Strongly agree	Agree	Neutral	Disagree	Strongly disagree
1	2	3	4	5

8) Do you have any questions about your medicines which have not been answered? If yes, please list.

9) I feel that I was encouraged to have a say in decisions about my medicines

Strongly agree	Agree	Neutral	Disagree	Strongly disagree
1	2	3	4	5

10) Have you seen a pharmacist during your time in hospital? (Multiple choice – single answer)

☐ Yes
☐ No or not sure

Paper surveys:
If yes, please answer questions 11 and 12 below.
If no, please skip to question 13.

Qualtrics surveys:
Branching logic - automatically skips to question 13.

11) There was good communication between the pharmacist and doctor about my medicines
(On Qualtrics this question was only displayed to patients to answered yes to Question 10)

Strongly agree	Agree	Neutral	Disagree	Strongly disagree	Unsure
1	2	3	4	5	6

12) I felt that any questions or concerns I had about my medicines were answered by the pharmacist
(On Qualtrics this question was only displayed to patients to answered yes to Question 10)

Strongly agree	Agree	Neutral	Disagree	Strongly disagree
1	2	3	4	5

Some patients are having their medicines written on the medicine chart by pharmacists (also known as medicine charting) after the pharmacist and the doctor have worked together to understand the patient's needs and goals and worked out what medicines are needed to best care for the patient.

The following questions are relating to your opinions on this new way of providing care.

13) What do you think are the benefits of having a pharmacist write your medicines on the medicine chart? Please list.

14) Are there any reasons why a pharmacist should not write your medicines on the medicine chart? Please list.

15) I trust a pharmacist to ensure I get the right medicines when I am in hospital

Strongly agree	Agree	Neutral	Disagree	Strongly disagree
1	2	3	4	5

16) I believe pharmacists have the appropriate knowledge and training to write my medicines on the medicine chart

Strongly agree	Agree	Neutral	Disagree	Strongly disagree
1	2	3	4	5

17) believe that a doctor should check the medicine chart written by a pharmacist before I am given the medicines

Strongly agree	Agree	Neutral	Disagree	Strongly disagree
1	2	3	4	5

18) I <u>do not</u> believe pharmacists should be involved in writing medicines on the medicine chart

Strongly agree	Agree	Neutral	Disagree	Strongly disagree
1	2	3	4	5
